# Cytotoxic Effects on Breast Cancer Cell Lines of Chalcones Derived from a Natural Precursor and Their Molecular Docking Analysis

**DOI:** 10.3390/molecules27144387

**Published:** 2022-07-08

**Authors:** Luis Bustos, Carlos Echiburú-Chau, Alejandro Castro-Alvarez, Ben Bradshaw, Mario J. Simirgiotis, Marco Mellado, Claudio Parra, Mauricio Cuellar

**Affiliations:** 1 Laboratorio de Química Orgánica y Productos Naturales, Facultad de Ciencias Agronómicas, Universidad de Tarapacá, Av. General Velásquez 1775, Arica 1000000, Chile; luisbustosg@gmail.com (L.B.); c_echiburu@hotmail.com (C.E.-C.); 2 Departamento de Ciencias Preclínicas, Facultad de Medicina, Universidad de La Frontera, Av. Francisco Salazar 01145, Temuco 4780000, Chile; alejandro.castro.a@ufrontera.cl; 3 Laboratori de Química Orgánica, Facultat de Farmàcia, IBUB, Universitat de Barcelona, Av. Joan XXIII, s/n, 08028 Barcelona, Spain; benbradshaw@ub.edu; 4 Instituto de Farmacia, Facultad de Ciencias, Universidad Austral de Chile, Valdivia 5110566, Chile; mario.simirgiotis@uach.cl; 5 Instituto de Investigación y Postgrado, Facultad de Ciencias de la Salud, Universidad Central de Chile, Santiago 8330507, Chile; marco.mellado@pucv.cl; 6 Centro de Investigación Farmacopea Chilena, Escuela de Química y Farmacia, Facultad de Farmacia, Universidad de Valparaíso, Av. Gran Bretaña 1093, Valparaíso 2360102, Chile

**Keywords:** antioxidant, bioactive compounds, breast cancer, cytotoxic, molecular docking, *Senecio nutans*, synthesis

## Abstract

This study aimed to determine the in vitro cytotoxicity and understand possible cytotoxic mechanisms via an in silico study of eleven chalcones synthesized from two acetophenones. Five were synthesized from a prenylacetophenone isolated from a plant that grows in the Andean region of the Atacama Desert. The cytotoxic activity of all the synthesized chalcones was tested against breast cancer cell lines using an MTT cell proliferation assay. The results suggest that the prenyl group in the A-ring of the methoxy and hydroxyl substituents of the B-ring appear to be crucial for the cytotoxicity of these compounds. The chalcones 12 and 13 showed significant inhibitory effects against growth in MCF-7 cells (IC_50_ 4.19 ± 1.04 µM and IC_50_ 3.30 ± 0.92 µM), ZR-75-1 cells (IC_50_ 9.40 ± 1.74 µM and IC_50_ 8.75 ± 2.01µM), and MDA-MB-231 cells (IC_50_ 6.12 ± 0.84 µM and IC_50_ 18.10 ± 1.65 µM). Moreover, these chalcones showed differential activity between MCF-10F (IC_50_ 95.76 ± 1.52 µM and IC_50_ 95.11 ± 1.97 µM, respectively) and the tumor lines. The in vitro results agree with molecular coupling results, whose affinity energies and binding mode agree with the most active compounds. Thus, compounds **12** and **13** can be considered for further studies and are candidates for developing new antitumor agents. In conclusion, these observations give rise to a new hypothesis for designing chalcones with potential cytotoxicity with high potential for the pharmaceutical industry.

## 1. Introduction

Despite substantial advances in early detection and treatment, breast cancer is a critical global public health problem, the second leading cause of death in women [1,2]. Furthermore, chemotherapeutic agents traditionally used to treat cancer have high toxicity, multidrug resistance, and lack of selectivity, limiting their effectiveness [3,4]. So, the search and development of new drugs play an essential role in cancer control. Ideally, any anticancer drug should exert a cytotoxic effect on malignant cells with a minimal impact on normal cells. Most drugs developed to combat cancer are of natural origin or inspired by them [5]. The compounds isolated from natural sources have a relevant role in developing new palliative therapies.

**Figure 1:**
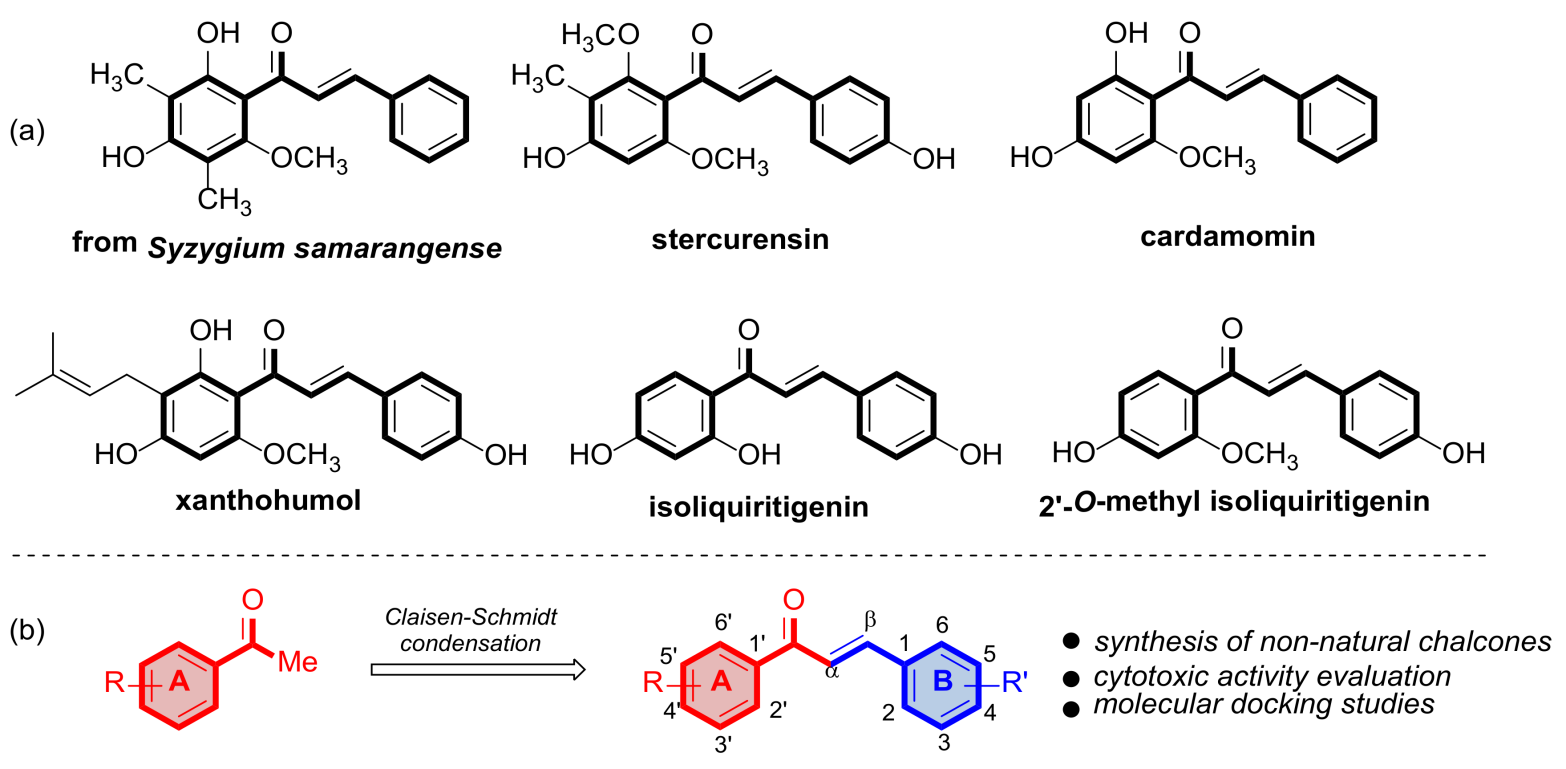
(**a**) Chalcones of natural origin with antiproliferative activity in various cancer lines. (**b**) Strategy to synthesize chalcones using a Claisen–Schmidt reaction.

In this context, chalcones and their derivatives maintain constant interest among scientists due to their broad spectrum of pharmacological activities, such as anticancer properties [6,7], among others. Chalcones, in their structure, have two aromatic rings linked by a three-carbon α, β-unsaturated carbonyl system and are fundamental, intermediate compounds in the biosynthesis of flavonoids and isoflavonoids in plants. Synthetic and natural chalcones are considered pharmacologically important compounds [8]. Numerous chalcones have been isolated from natural sources and have been shown to affect each level of carcinogenesis and exhibit activity against cancer cells (Figure 1). Amor et al. isolated the compound 2,4-dihydroxy-6-methoxy-3,5-dimethylchalcone from *Syzygium samarangense* (Myrtaceae) and determined that this compound has antiproliferative activity in MCF-7 and SKBR-3 [9]. Other naturally occurring compounds that show antiproliferative activity in cancer cell lines (SW-480) are estercurensin and cardamomine [10]. Chalcone-type compounds, such as xanthohumol, a compound isolated from *Humulus lupulus* (Cannabaceae), have growth-inhibitory effects on prostate (PC-3 and DU-145) and cervical (HeLa) cancer cells [11,12]. Other chalcones that exhibit antiproliferative activity in cancer cells are isoliquiritigenin and 2′-*O*-methyl isoliquiritigenin [13]. In this context, natural products have relatively high safety profiles. However, in most cases, the cytotoxic effect, chemical stability, oral bioavailability, and metabolic stability of natural compounds are far from synthetic compounds.

Given the need for new drugs with high selectivity, low toxicity, and good metabolic stability against breast cancer, prenylated acetophenone **1b**, isolated from a plant that grows in the Andean region of the Atacama Desert, was used as starting material to obtain chalcones. This prenylated acetophenone has demonstrated good cytotoxic activity [14], suggesting that synthesized chalcones based on this scaffold could potentially have better activity and selectivity than their precursor. As far as we know, there is no information on the cytotoxic activity of chalcones that contain **1b** in their A ring in their structure. Therefore, this study aims to determine the in vitro cytotoxicity and understand the possible cytotoxic mechanisms through in vitro and in silico study from chalcones synthesized from a naturally occurring acetophenone.

## 2. Results and Discussion

### 2.1. Cytotoxic Activity of Chalcone Compounds against Breast Cancer

**Scheme 1:**
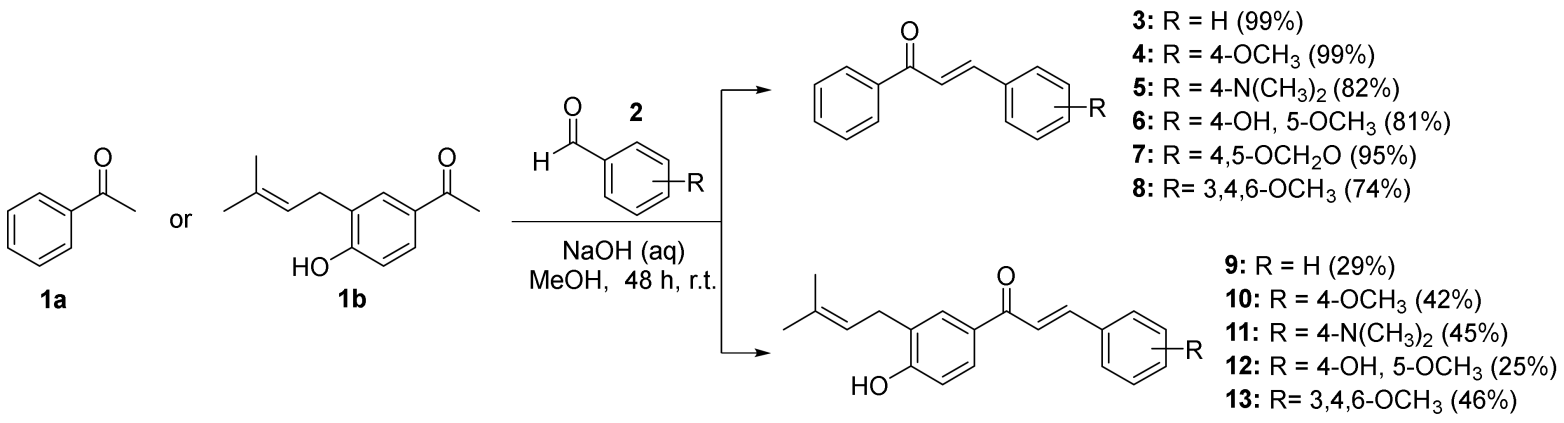
Synthesis of chalcone compounds via Claisen–Schmidt condensation.

This study continues the search for biologically active compounds from a naturally occurring prenyl acetophenone [15,16]. However, to expand our knowledge about chalcones and how their substituents influence their bioactivity, we will use 11 compounds that we have previously synthesized from readily available starting materials (Scheme 1).

The cytotoxic activity of all the synthesized chalcones was tested against breast cancer cell lines using an MTT cell proliferation assay. All compounds were tested in vitro at 10 and 100 µM using the cell viability assay in MCF-7 for 24 h in normoxia (Table 1). Firstly, the cytotoxic effect of the chalcone compounds modified in ring B and with an unsubstituted ring A was tested (compounds **3**–**8**). As shown in Table 1, compounds **5**, **6**, and **7** were the most interesting, with a cell viability percentage between 47–42% in the treatment at 10 µM in MCF-7. Secondly, compounds with modifications in the A and B rings (**9**–**13**) were tested. The substitution of the A-ring with a hydroxyl group at C-4′ and a prenyl group at C-5′ shows a potentiating effect on cytotoxic activity compared to unsubstituted chalcones in the A-ring. Furthermore, it was observed that the substitution with a methoxy group at C-5 in the B-ring was beneficial for cytotoxic effects, as indicated by percentage values of **3** (81.3%) vs. **4** (74.5%) and **9** (93.8%) vs. **10** (73.7%).

**Figure 2:**
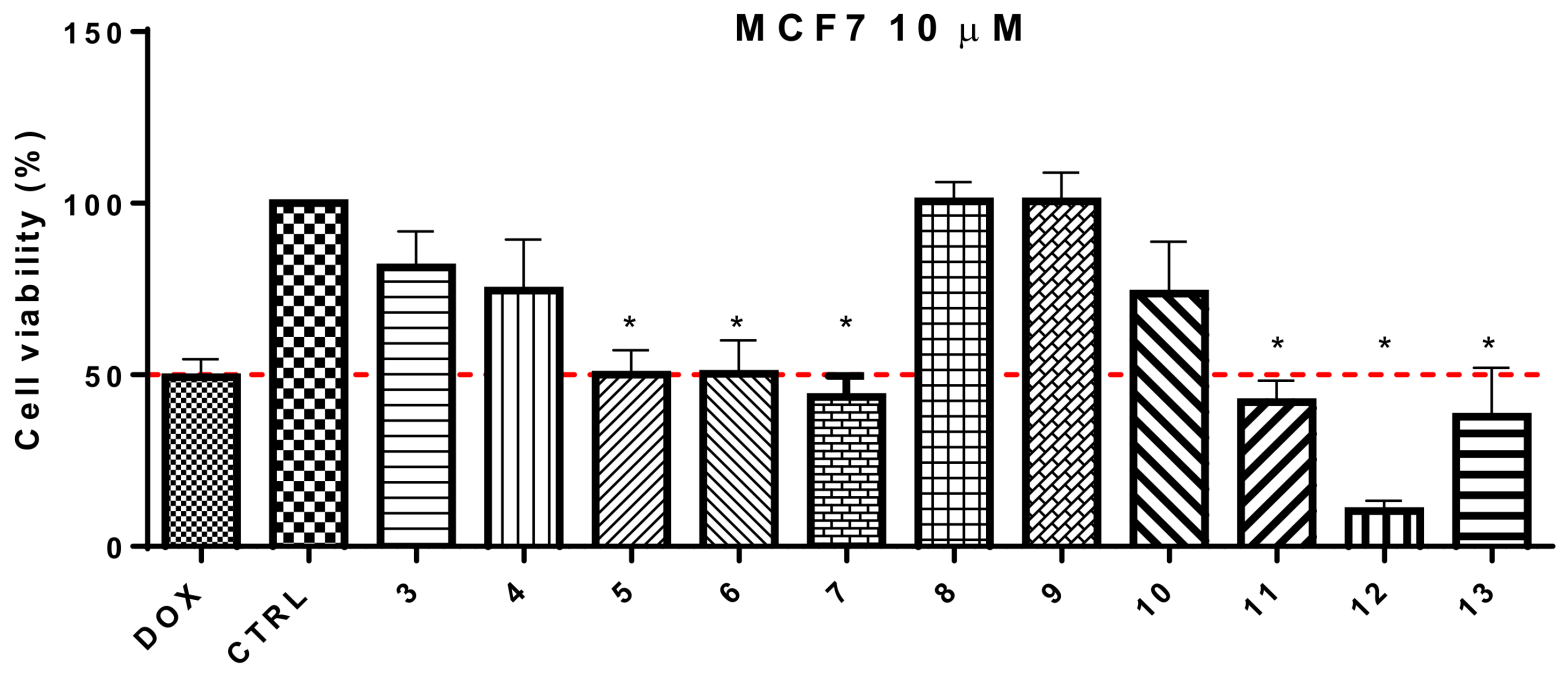
Cell viability of the synthesized chalcones with 10 µM treatments in MCF-7 cells. *—cell viability ≤ 50%.

Compound 12 bearing a methoxy group at C-5′ and hydroxyl at C-4 of the B-ring showed a greater cellular growth inhibition effect (10.3%). However, the results also suggested that compounds bearing a nitrigen (such as –N(Me)_2_; 42.0%) at the C-4 site of the B-ring could be of interest [17]. Moreover, we also found that the introduction of methoxy groups on B-ring in C-3, C-4, and C-6 sites decreased cell viability (**13**). Because of the results, we considered changing the hydroxy group of the A-ring for a methoxy group to evaluate the activity. However, all the synthesized compounds did not show relevant cytotoxic activity. According to the result in Figure 2, the compounds that exhibited more promising cytotoxicity in MCF-7 cells (cell viability ≤ 50%) are marked with an asterisk.

Table 2 shows IC_50_ of the most active chalcones in different breast cancer lines. Compounds **5** and **7** do not show significant activity relative to the other compounds. The compound **6** (21.55 ± 2.71 µM) was more active than the starting material **1b** (79.40 ± 12.25 µM), and it is recognized for its cytotoxic activity in cells MCF-7 [14]. Moreover, this chalcone showed slight differential activity between MCF-10F (72.60 ± 10.31 µM) and the tumor lines. However, the prenylated chalcones were most active in all breast cancer lines than chalcones without this substituent. The compounds **12** and **13** possess a similar activity in the MCF-7 cells (4.19 ± 1.04 µM and 3.30 ± 0.92 µM) and ZR-75-1 cells (9.40 ± 1.74 µM and 8.75 ± 2.01 µM), but the MDA-MB-231 cells show slightly different inactivity (6.12 ± 0.84 µM and 18.10 ± 1.65 µM). Compounds **12** and **13** presented IC_50_ values that make them highly cytotoxic compounds against all cell lines with a differential effect.

**Figure 3:**
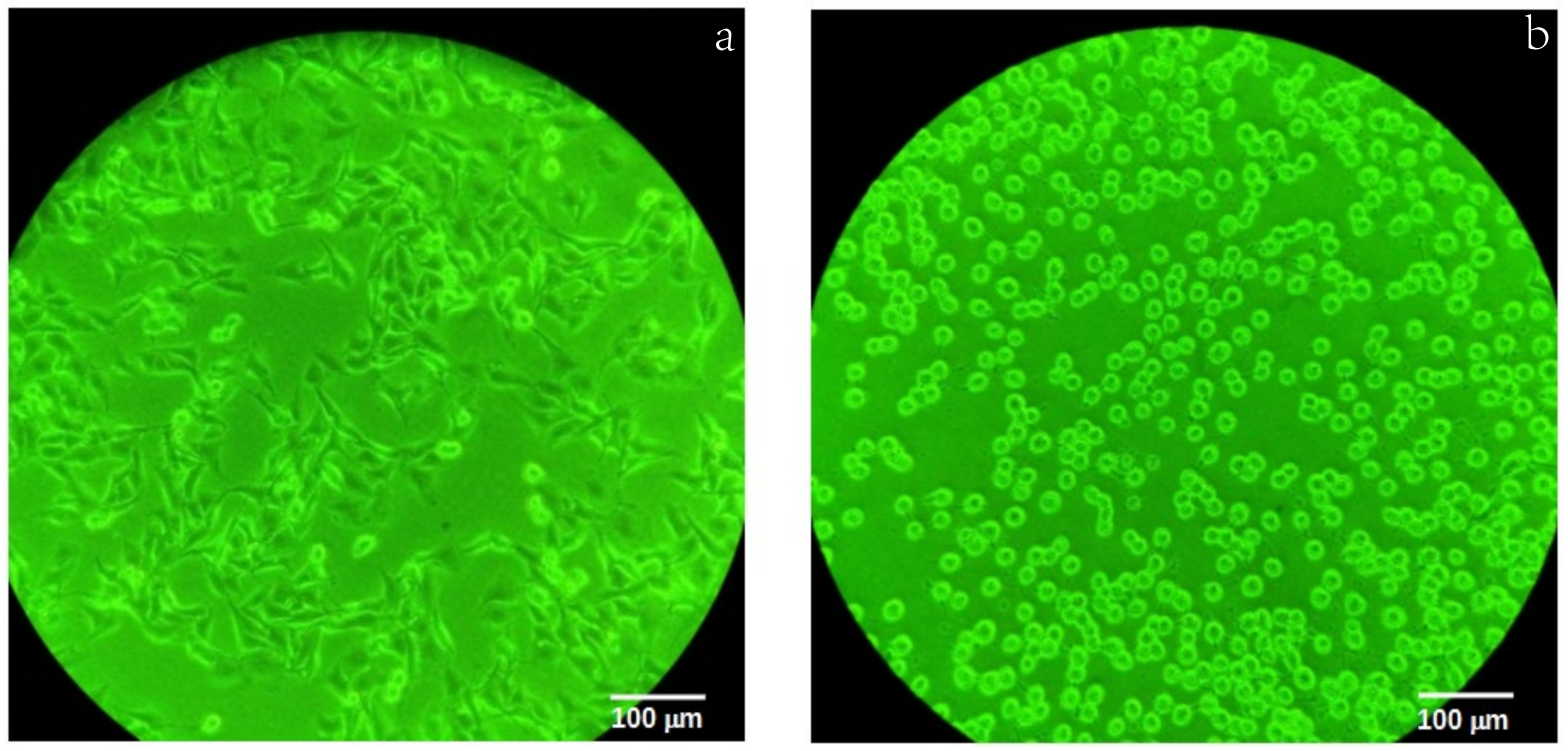
Change in the morphology of MDA-MB-231 cells at (**a**) 0 h and (**b**) after 6 h of incubation after addition of compound 6.

Among the tested chalcones, the hydroxyl group in ring A is essential for cytotoxicity, as has been studied in various biological assays, predominantly for its anticancer properties [18,19]. The presence of electron-withdrawing and donor groups affects the α,β-unsaturated system [18,20], which in turn affects cytotoxicity. Additionally, the prenyl group increases lipophilicity, improves membrane binding, and increases transmembrane transport, and these characteristics favor increased biological activity [12,21]. Besides, this study observed the speed with which the compounds act on cells, causing their death (see Figure 3).

On the other hand, compounds **6** and **12** share the B-ring’s structure, similar to that found in ferulic acid. This substitution could increase biological activity since this acid decreases cell viability, induces apoptosis, and suppresses potential metastasis in MDA-MB-231 cells [22]. Moreover, the trimethoxyphenyl substitution presented by chalcone **13** improves the cytotoxic activity on all cells except on MDA-MB-231 compared to compound **12**, so that the portion derived from ferulic acid would have a better effect on the activity. Chalcones **12** and **13** presented IC_50_ values higher than those reported by the chalcone compounds synthesized by Abosalim et al. as possible anticancer agents [23]. Furthermore, these chalcones were more active in the MCF-7 and MDA-MB231 cell lines than the compounds synthesized by Patel et al. [24]. Moreover, the chalcones selected in this work showed significant cytotoxicity against these cancer cell lines with an IC_50_ between 4 to 3 μM on the growth of the MCF-7 cells, which resulted in better results than Paratocarpin E for the same cell lines (19.6 μM) [25].

### 2.2. Understanding the Possible Cytotoxic Mechanism, an In Silico Study

Ng et al. correlated the cytotoxic activity of a series of chalcones with dihydrofolate reductase (DHFR) [26,27]. Based on that evidence, molecular docking was performed at the catalytic site of this enzyme with compounds **6**, **12**, and **13**. The results in Table 3 show an affinity trend for DHFR similar to the inhibition trend shown in Table 2. We can see these effects in the binding energy (∆G Bind), where compound **13** is the most active with −59.16 kcal/mol, compound **12** with −54.66 kcal/mol, and compound **6** with −51.29 kcal/mol. All three compounds have indispensable chalcone A-ring interactions such as hydrogen bonding with Ala9 and carbonyl group, hydrophobic interactions with Leu22, and π-stacking between phenone group and Phe34 residue.

**Figure 4:**
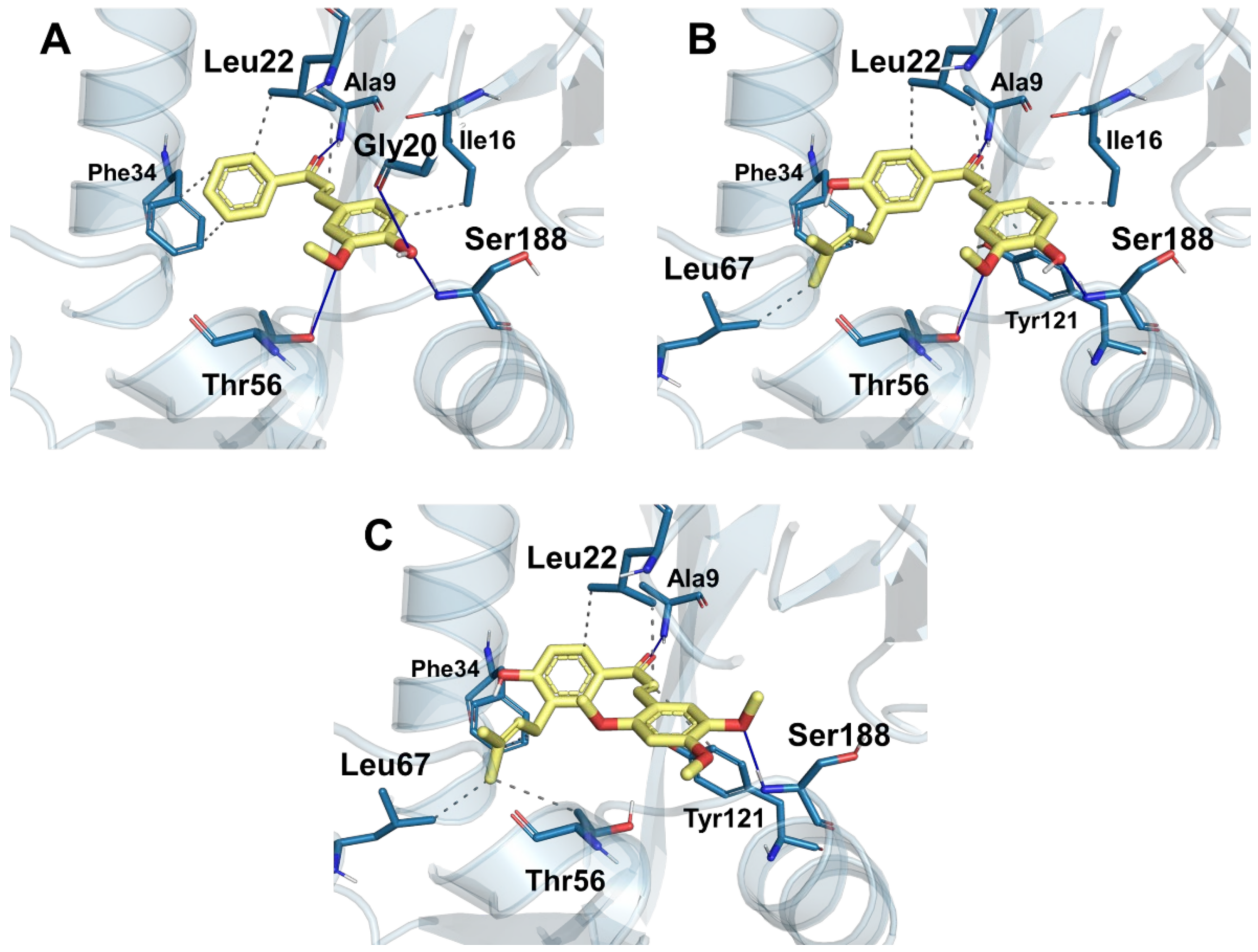
Interactions of the synthesized chalcones with the catalytic site of DHFR. (**A**) Binding mode of compound **6** and most residues represented. (**B**) Binding mode of compound **12** and most residues represented. (**C**) Binding mode of compound **13** and most residues represented.

Compound **6** has better ∆G Coulomb (−21.50 kcal/mol) due to the interaction with the hydroxyl (phenyl B) with residues Gly20 and Ser188; in addition, there is a second hydrogen bond with Thr56 and the methoxyl (Figure 4A). Compound **12** has higher hydrogen bonding energy (∆G HBond −2.04 kcal/mol) and more significant lipophilic (∆G Lipo −23.80 kcal/mol) and van der Waals (∆G vdW −48.46 kcal/mol) characteristics; this can be explained by the 3-methylbutenyl (prenyl group) radical interacting favorably with residues Leu67 (Figure 4B). These same characteristics can be seen in compound **13**, where the difference in binding energy is due to the methoxyl substituents of the chalcone B-ring, which causes ligand displacement, enhancing hydrophobic interactions with the Leu67 and Thr56 residues (Figure 4C); we can see this in the ∆G Lipo energy of −27.34 kcal/mol and the ∆G vdW energy of −53.45 kcal/mol, which are the highest compared to the others.

## 3. Materials and Methods

### 3.1. Chemistry

All reactions were carried out under anhydrous conditions in an argon atmosphere with dry, freshly distilled solvents. Analytical thin-layer chromatography was performed on SiO_2_ (Merck silica gel 60 F_254_), and the spots were located with 1% aqueous KMnO_4_. Chromatography, referring to flash chromatography, was carried out on SiO_2_ (SDS silica gel 60 ACC, 35–75 mm, 230–240 mesh ASTM). Drying of organic extracts during workup of reactions was performed over anhydrous MgSO_4_ except where stated otherwise. Evaporation of solvent was accomplished with a rotatory evaporator. NMR spectra were recorded in CDCl_3_ or MeOD on a Varian VNMRS 400 (Varian, Palo Alto, CA, USA). Chemical shifts of ^1^H and ^13^C NMR spectra are reported in ppm downfield (δ) from Me_4_Si. Electron-spray ionization mass spectra in positive mode (ESI-MS) data were recorded on a Bruker Esquire 3000 + spectrometer (Bruker Daltonics Inc., Billerica, MA, USA).

### 3.2. Synthesis of Chalcones

To the mixture of a commercial benzaldehyde (1.2 mmol), acetophenone **1a** or **1b** (1.22 and 2.08 mmol) in MeOH (5 mL), a NaOH saturated solution, was added (in 10 mL of methanol), and the mixture was stirred for 48 h at room temperature. After the disappearance of the reactant (TLC), 5% HCl solution was added until pH ~7 to end the reaction, the mixture was extracted with EtOAc (3 × 50 mL), and the combined organic layers were dried (anhydrous Na_2_SO_4_). Removal of the solvent and purification of the residue by column chromatography or recrystallization gave the target products.

### 3.3. Characterizacion Data

(E)-1-(4-hydroxy-3-(3-methylbut-2-en-1-yl)phenyl)-3-(4-hydroxy-3-methoxyphenyl)prop-2-en-1-one (**12**). *Yellow solid (25% yield). ^1^H NMR (400 MHz, MeOD) δ: 7.85 (1H, m, H6), 7.84 (1H, s, H2), 7.67 (1H, d, J = 15.5 Hz, H_β_), 7.56 (1H, d, J = 15.5 Hz, H_α_), 7.18 (1H, dd, J = 8.1, 1.8 Hz, H6′), 6.85 (1H, d, J = 8.1 Hz, H5), 6.83 (1H, d, J = 8.1 Hz, H5′), 5.34 (1H, ddt, J = 9.1, 7.4, 1.3 Hz, H2″), 3.92 (3H, s, OCH_3_), 3.33 (2H, d, J = 7.4 Hz, CH_2_), 1.74 (3H, s, CH_3_), 1.73 (3H, s, CH_3_). ^13^C NMR (100 MHz, MeOD) δ: 191.0 (C=O), 161.6 (C4), 150.8 (C4′), 149.4 (C3′), 145.9 (H_β_), 133.7 (C3″), 131.7 (C2), 131.1 (C1), 129.9 (C6), 129.8 (C3) 128.5 (C1′), 124.7 (C6′), 123.4 (C2″), 120.0 (C_α_), 116.5 (C5′), 115.5 (C5), 112.1 (C2′), 56.5 (OCH_3_), 29.2 (C1″), 26.0 (C4″), 17.9 (C5″). EI-MS: m/z 339.1556 [M^+^] (100)*.

### 3.4. Cell Culture

The cell culture methodology was used with modifications [14]. Three human breast cancer cell lines were used for this study: MCF-7 (ATCC^®^ HTB-22™), ZR-75-1 (ATCC^®^ CRL-1500™), MDA-MB-231 (ATCC^®^ HTB-26™), and the non-tumorigenic MCF-10F (ATCC^®^ CRL-10318™) cell lines. Cells were cultured in specific media according to ATCC recommendations. The incubation condition was established at 37 °C, a complete humid atmosphere, 5% CO_2_ and 95% O_2_.

### 3.5. Cytotoxic Assays

The cytotoxic effect of the chalcone compounds was assessed in MCF-10F cells in a dose- and time-dependent manner [14]. Cells (1 × 10^4^ and 4 × 10^4^) cultured under normoxic conditions were seeded in 24-well plates in quadruplicate and incubated for 4 days until 70% confluence. After this incubation period, cells were exposed to concentrations of the synthesized compounds dissolved in ethanol (50%) at concentrations ranging from 0 to 96.8 µg/mL (dissolved in 0.5% DMSO). Doxorubicin was used as the control drug, and for all data a *p* < 0.05 value was considered statistically significant. Cell viability was assessed using a neutral red uptake assay after 24 h of treatment.

### 3.6. Molecular Docking

Molecular docking studies were performed to understand the binding modes of the synthesized compounds to the dihydrofolate reductase protein using the Gilde software [28]. This program allows for determining the most suitable positions of the ligands in the binding site of interest of the protein, considering the total flexibility of the ligand [29].

The preparation for the protein (PDB ID: 4M6J; resolution 1.2 Å [30]) was minimized to a gradient of 0.01 kcal/mol Å. The default parameters in the Glide docking program were: 5000 poses per ligand for the initial docking phase; standard precision (SP) method; RMS deviation less than 5.0 Å; and maximal atomic displacement less than 1.3 Å. The binding site was defined as a cubic region (Grid) encompassing all protein atoms from the co-crystallized ligand (NADPH) within 15.0 Å. The three compounds’ structures were built using the 2D sketcher module of the Schrodinger Suite, and ligand preparation was performed using the LigPrep module [31]. For the five best poses achieved with the described procedure, Induced Fit Docking was performed in order to improve the interactions between the synthesized ligands and the receptor [32,33]; the same Grid parameters were maintained with a refinement of the residues at 6.0 Å distance from the ligand poses and a rescoring of the best poses achieved with the eXtra Precision (XP) method [34]. Finally, the ligand-binding energies and ligand strain energies for the chalcones were estimated using Prime MM-GBSA (Molecular Mechanics/Generalized Born Model and Solvent Accessibility), which includes the OPLS4 force field [35], VSGB solvent model [36,37], and rotamer search algorithms. The MM/GBSA method is used to calculate the relative binding affinity of ligands to the receptor (in kcal/mol). Because the MM/GBSA [38] binding energies are estimates of binding free energies, a lower number indicates greater binding. The energies obtained for the complexes were estimated automatically using the energy terms and equation systems presented in the following.

$$\Delta G_{bind}=G_{complex}-\left(G_{receptor}-G_{ligand}\right)$$

$$\Delta G_{bind}=\Delta E_{MM}+\Delta G_{GB}+\Delta G_{SA}$$

$$\Delta G_{bind}=\Delta G_{Coulomb}+\Delta G_{Hbond}+\Delta G_{Lipo}+\Delta G_{vdW}+\Delta G_{Packing}+\Delta G_{SolvGB}$$

where Δ*G_bind_* represents the total binding free energy upon ligand–receptor binding; Δ*E_MM_* is the total gas phase energy in the molecular mechanics (*MM*) and force field (OPLS4); Δ*G_Coulomb_*, Δ*G_Packing_*, Δ*G_Lipo,_* and Δ*G_vdW_* correspond, respectively, to the electrostatic, π-π packing correction, lipophilic and van der Waals energies; and Δ*G_SolvGB_* is the polar electrostatic solvation energy calculated via the generalized Born (*GB*) method.

### 3.7. Statistical Analysis

All data were analyzed using OriginPro 9.1 software packages (Originlab Corporation, Northampton, MA, USA). Significant difference comparisons were made by the Tukey test; the statistical significance was defined as *p* ≤ 0.05.

## 4. Conclusions

The results presented in this study suggest that the synthesized chalcones **12** and **13** showed significant inhibitory effects against the growth of human cancer cell lines in vitro. Both compounds showed differential activity between non-tumor and the tumor lines. Furthermore, the prenyl group appears to be crucial for the cytotoxicity of these compounds. This evidence coincides with the molecular docking results, whose affinity energies and binding mode agree with the most active compounds (compounds **12** and **13**) due to enhanced binding of the methoxyl groups of the A-ring via hydrogen bond formation and enhanced hydrophobic interactions of the prenyl group with the amino acids Leu67 and Thr56. These observations give rise to a new hypothesis for designing new chalcones with potential antineoplastic activity. Therefore, these molecules based on the natural compound **1b** scaffold represent good lead compounds in the search for new pharmaceuticals to fight breast cancer.

## Figures and Tables

**Table 1:** 10 and 100 µM treatments in MCF-7 cells with the synthesized chalcones.

Compound	MCF-7 10 µM	MCF-7 100 µM
3	81.3 ± 9.4	65.2 ± 5.7
4	74.5 ± 10.3	55.1 ± 5.1
5	47.6 ± 3.2	52.4 ± 4.3
6	47.0 ± 4.9	4.9 ± 0.6
7	43.5 ± 3.4	38.6 ± 4.3
8	100.5 ± 12.6	94.4 ± 11.6
9	93.8 ± 7.9	82.5 ± 6.1
10	73.7 ± 6.7	62.3 ± 7.6
11	42.0 ± 4.3	42.4 ± 3.9
12	10.3 ± 0.6	3.6 ± 0.9
13	32.4 ± 4.5	3.1 ± 0.4
Doxorubicin	49.3 ± 2.2	57.6 ± 2.9

Doxorubicin was considered as a control drug (*n* = 3). The values were expressed as mean ± SD (*n* = 6).

**Table 2:** IC_50_ of synthesized chalcones in different lines of breast cancer.

Compounds	IC_50_ Cell Lines (µM)			
	MCF-10F	ZR-75-1	MCF-7	MDA-MB-231
1b	79.51 ± 9.90 ^a^	61.62 ± 14.09 ^a^	79.40 ± 12.25 ^a^	74.04 ± 8.72 ^a^
3	>80	>80	>80	>80
4	>80	>80	>80	>80
5	>80	76.51 ± 12.63 ^a^	>80	79.84 ± 9.31 ^a^
6	72.60 ± 10.31 ^a^	43.17 ± 6.58 ^b^	21.55 ± 2.71 ^b^	51.96 ± 4.53 ^b^
7	>80	>80	>80	>80
8	>80	>80	>80	>80
9	>80	>80	>80	>80
10	>80	>80	>80	77.98 ± 4.34 ^a^
11	78.43 ± 21.32 ^a^	78.19 ± 6.87 ^a^	>80	72.34 ± 2.33 ^a^
12	75.76 ± 11.52 ^a^	9.40 ± 1.74 ^c^	4.19 ± 1.04 ^c^	6.12 ± 0.84 ^d^
13	75.11 ± 11.97 ^a^	8.75 ± 2.01 ^c^	3.30 ± 0.92 ^c^	18.10 ± 1.65 ^c^
Doxorubicin ^1^	83.91 ± 8.99 ^a^	79.84 ± 10.95 ^a^	110.80 ± 8.95 ^a^	47.62 ± 5.74 ^b^

^1^ Doxorubicin was considered as a control drug (*n* = 3) The values were expressed as mean ± SD (*n* = 6). Values having different superscripts differ significantly (*p* < 0.05).

**Table 3:** Binding energies of the synthesized compounds.

Comp.	∆*G_Bind_*	∆*G_Coulomb_*	∆*G_Hbond_*	∆*G_Lipo_*	∆*G_Packing_*	∆*G_Solv GB_*	∆*G_vdW_*
6	−51.29	−21.50	−1.43	−20.16	−2.04	29.55	−39.46
12	−54.66	−19.76	−2.04	−23.80	−1.60	27.31	−48.46
13	−59.16	−18.80	−1.49	−27.34	−1.38	31.97	−53.45

## Data Availability

Data are contained within the article.

## Sample Availability

Samples of the compounds are not available from the authors.
